# Supplementary material for: Comparative genomics provides new insights into the diversity, physiology, and sexuality of the only industrially exploited tremellomycete: Phaffia rhodozyma
Source: BMC Genomics. 2016 Nov 9;17:901. doi: 10.1186/s12864-016-3244-7 (PMC5103461; doi:10.1186/s12864-016-3244-7)
Supplement: Additional file 6: — List of orphan genes with links to PFAM (related to Additional file 1: Table S1). (ZIP 1428 kb) [file 12864_2016_3244_MOESM6_ESM.zip › PFAM_UNIQUE/G05867_P.html]

#                                                                            --- full sequence --- -------------- this domain -------------   hmm coord   ali coord   env coord
  
# target name        accession   tlen query name           accession   qlen   E-value  score  bias   #  of  c-Evalue  i-Evalue  score  bias  from    to  from    to  from    to  acc description of target
  
#------------------- ---------- ----- -------------------- ---------- ----- --------- ------ ----- --- --- --------- --------- ------ ----- ----- ----- ----- ----- ----- ----- ---- ---------------------
  
G05867\_P             -            560 ATP-synt\_D           PF01813.12   196    0.0019   16.4   0.4   1   1   1.8e-06    0.0035   15.6   0.4   115   162    43    90    24    92 0.92 - ATP synthase subunit D  
G05867\_P             -            560 CENP-F\_leu\_zip       PF10473.4    140      0.15   10.6   6.0   1   1    0.0014      0.26    9.8   6.0    24    92    27    95    25    98 0.95 - Leucine-rich repeats of kinetochore protein Cenp-F/LEK1  
G05867\_P             -            560 DUF776               PF05604.6    178      0.19   10.6   2.9   1   2   0.00034         1    8.2   0.1    98   136   275   313   269   349 0.80 - Protein of unknown function (DUF776)  
G05867\_P             -            560 DUF776               PF05604.6    178      0.19   10.6   2.9   2   2     0.043   1.3e+02    1.4   0.6    32    77   483   530   465   537 0.69 - Protein of unknown function (DUF776)  
G05867\_P             -            560 DUF972               PF06156.8    107      0.14   11.2   1.7   1   1    0.0012      0.27   10.3   1.7     9    61    28    80    24    98 0.80 - Protein of unknown function (DUF972)
